# Invasive arterial blood pressure in the neonatal intensive care: a valuable tool to manage very ill preterm and term neonates

**DOI:** 10.1186/1824-7288-41-S1-A9

**Published:** 2015-09-24

**Authors:** Michelina Di Biase, Anna Casani, Luigi Orfeo

**Affiliations:** 1 Neonatal Intensive Care Unit, Maternal and Child Health Department, Rummo Hospital Benevento, Italy

## 

Blood pressure monitoring is essential in managing hemodynamically unstable neonates and preterm infants. Non-invasive blood pressure measurement (NIBP) with oscillometric technique is in widespread use in the Neonatal Intensive Care Units (NICUs). Nonetheless NIBP is not pretty accurate when compared with invasive monitoring since it generally over read mean blood pressure in particular when the infants are hypotensive so it falsely reassures neonatologists [1-4]. Invasive arterial blood pressure (IABP) methods is considered the gold standard for circulatory management of ill neonates [5]. Along with the more accuracy, IABP measurement has a number of advantages over NIBP, namely it allows beat-to-beat pressure measurement to closely monitor patients with very changeable conditions, arterial blood sampling is easily performed as well as cardiac stroke volume can be derived from characteristics of the arterial pressure pulse. The commonly used method is by means of an umbelical artery catheter, wherever possible, or by placing a cannula needle in a different artery, usually radial [5-8]; a column fluid directly connects the arterial system to a pressure transducer where the arterial pulse is converted into an electrical signal that in turn will be processed via a microprocessor, amplified and eventually displayed as the blood pressure waveform against time [5]. In order to ensure a reliable assessment of blood pressure nurses should be wary about one of the commonest sources of error, namely introduction of small air bubble in the system [5]. Thrombo-embolism, vasospasm, thrombosis, haemorrage and infection are complications of arterial cannulation [9]. Haematoma and peripheral nerve injury may also occur in case of peripheral cannulation. A close supervision by nurses encompasses observation for adequate patency of artery by monitoring hourly colour, temperature and perfusion of digits and limbs. Blanching, redness, cyanosis and changes in temperature must be quickly reported to the medical staff. Severe bleeding as result of disconnected arterial line required a strict monitoring as well. In addition nursing management consists in performing level and zero arterial line at the beginning of every shift and every time the neonate is turned or moved. The heparinized salin infusion should be changed every 24 hours and the infusion line every third day [10-15].

In conclusion invasive arterial blood pressure technique, if correctly performed by neonatologists and closely monitored by nurses, represents a valuable tool to tailor treatment in very ill preterm neonates.
